# Supplementary material for: Resolving the full spectrum of human genome variation using Linked-Reads
Source: Genome Res. 2019 Apr;29(4):635–45. doi: 10.1101/gr.234443.118 (PMC6442396; doi:10.1101/gr.234443.118)
Supplement: Supplemental Material [file supp_gr.234443.118_Supplemental_longranger-2.2.zip › longranger-2.2.tar.bz2!/lr2.2-public/lib/stan/cmdstan/stan/doc/api/html/structstan_1_1gm_1_1range.html]

Stan: stan::gm::range Struct Reference


|  |  |
| --- | --- |
|  | Stan  2.5.0  probability, sampling & optimization |


- Main Page
- Related Pages
- Namespaces
- Classes
- Files

- Class List
- Class Index
- Class Hierarchy
- Class Members

All Classes Namespaces Files Functions Variables Typedefs Enumerations Enumerator Friends Macros Pages

- stan
- gm
- range

Public Member Functions |
Public Attributes |
List of all members

stan::gm::range Struct Reference

`#include <ast.hpp>`

|  |  |
| --- | --- |
| Public Member Functions | |
|  | range () |
|  | |
|  | range (expression const &low, expression const &high) |
|  | |
| bool | has\_low () const |
|  | |
| bool | has\_high () const |
|  | |

|  |  |
| --- | --- |
| Public Attributes | |
| expression | low\_ |
|  | |
| expression | high\_ |
|  | |

## Detailed Description

Definition at line 401 of file ast.hpp.

## Constructor & Destructor Documentation

|  |  |  |  |  |
| --- | --- | --- | --- | --- |
| stan::gm::range::range | ( |  | ) |  |

Definition at line 931 of file ast\_def.cpp.

|  |  |  |  |
| --- | --- | --- | --- |
| stan::gm::range::range | ( | expression const & | *low*, |
|  |  | expression const & | *high* |
|  | ) |  |  |

Definition at line 932 of file ast\_def.cpp.

## Member Function Documentation

|  |  |  |  |  |
| --- | --- | --- | --- | --- |
| bool stan::gm::range::has\_high | ( |  | ) | const |

Definition at line 940 of file ast\_def.cpp.

|  |  |  |  |  |
| --- | --- | --- | --- | --- |
| bool stan::gm::range::has\_low | ( |  | ) | const |

Definition at line 937 of file ast\_def.cpp.

## Member Data Documentation

expression stan::gm::range::high\_

Definition at line 403 of file ast.hpp.

expression stan::gm::range::low\_

Definition at line 402 of file ast.hpp.

---

The documentation for this struct was generated from the following files:

- src/stan/gm/ast.hpp
- src/stan/gm/ast\_def.cpp


---

[ Stan Home Page ]

*© 2011–2014,
Stan Development Team.*
